# Trapped Fourth Ventricle in a Pediatric Patient With a History of Post‐Traumatic Hydrocephalus and Ventriculoperitoneal Shunt: A Case Report and Literature Review

**DOI:** 10.1002/ccr3.72748

**Published:** 2026-06-10

**Authors:** Muhammad Hamza, Muhammad Ibrahim, Ihsan Ullah, Rehmat Farhaj, Fazeela Bibi, Khalil El Abdi, Bilal Aslam, Vohra Maham Hassan, Said Hamid Sadat

**Affiliations:** ^1^ Saidu Medical College Swat Pakistan; ^2^ Bannu Medical College Bannu Pakistan; ^3^ Khyber Medical University Peshawar Pakistan; ^4^ Jinnah Medical and Dental College Karachi Pakistan; ^5^ Faculty of Medicine and Pharmacy of Rabat Mohammed V University Rabat Morocco; ^6^ University of Lahore Lahore Pakistan; ^7^ Kabul University of Medical Sciences, Abu Ali Ibn Sina Kabul Afghanistan

**Keywords:** aqueductoplasty, arachnoid dissection, hydrocephalus, isolated fourth ventricle, trapped fourth ventricle, ventriculoperitoneal shunt

## Abstract

Trapped fourth ventricle (TFV) is a rare, serious complication of ventriculoperitoneal (VP) shunting that can mimic posterior fossa tumors and cause life‐threatening brainstem compression. We report the case of a 7‐year‐old boy with a history of a VP shunt for post‐traumatic hydrocephalus who presented with progressive drowsiness and gait disturbances. While initial CT imaging suggested a tumor, subsequent MRI confirmed a markedly dilated fourth ventricle, diagnosing TFV. The patient underwent open posterior fenestration with microsurgical arachnoid adhesiolysis, which successfully restored cerebrospinal fluid dynamics. This intervention resulted in the complete resolution of his neurological symptoms at 1‐month follow‐up. This case highlights the importance of suspecting TFV in shunted patients with new neurological deficits and demonstrates that open fenestration is a highly effective treatment strategy, particularly when dense adhesions are clinically suspected. Timely diagnosis with advanced imaging and prompt surgical intervention are crucial to prevent irreversible damage and ensure favorable neurological outcomes.

## Key Clinical Message

Trapped Fourth Ventricle (TFV) requires high suspicion in shunted patients with new neurological deficits, as it can mimic posterior fossa tumors. Advanced imaging, particularly MRI, is essential for accurate diagnosis. Prompt surgical intervention is critical to prevent irreversible brainstem compression and ensure favorable outcomes.

## 1 Introduction

Hydrocephalus is a neurological condition marked by abnormal cerebrospinal fluid (CSF) accumulation, causing ventricular enlargement and potential brain damage [1, 2]. It results from CSF overproduction, flow obstruction, or impaired absorption, presenting with headaches, nausea, visual disturbances, and cognitive decline, which is diagnosed via CT or MRI [3]. Ventriculoperitoneal (VP) shunts, which divert CSF to the peritoneum, treat various forms of hydrocephalus, including congenital, tumor‐related, and normal pressure hydrocephalus [4]. However, VP shunts carry 2%–20% complication risks like infection, hemorrhage, and obstruction [4]. A rare but serious complication of VP shunt is trapped fourth ventricle (TFV), also referred to in the literature as an isolated fourth ventricle (IFV), where CSF accumulates due to aqueductal and foraminal blockages [5, 6], which may be discovered incidentally during imaging studies [7]. This condition can develop insidiously, sometimes years after the initial shunt placement, and is typically seen in patients with multiple shunt surgeries, potentially causing brainstem compression and neurological deficits [8, 9]. Symptomatic TFV requires surgical intervention [10]. The choice of surgical approach remains subject to institutional resources and expertise, with endoscopic techniques emerging as the preferred modality in well‐equipped centers [11]. Early recognition of TFV and intervention are critical to prevent irreversible neurological deficits. We present a case of TFV in a pediatric patient with a history of post‐traumatic hydrocephalus, initially misdiagnosed as a space‐occupying lesion.

## 2 Case History

A 7‐year‐old boy from Peshawar, Pakistan, presented to our outpatient department with a two‐week history of progressive drowsiness, gait disturbances, intermittent fever, and episodic loss of consciousness accompanied by irrelevant mumbling. His parents also reported sleep irregularities and behavioral changes.

The patient had a significant past surgical history. At age three, he underwent surgical repair of a depressed skull fracture following a fall. He recovered and remained asymptomatic for 2 years. At age five, he developed postprandial vomiting and a sudden loss of consciousness, leading to a diagnosis of post‐traumatic hydrocephalus. A ventriculoperitoneal (VP) shunt was placed at that time. Following shunt insertion, he made a full recovery and remained neurologically stable for 2 years until the onset of the current symptoms.

Upon examination, his Glasgow Coma Scale (GCS) score was 13/15 (E4V3M5), with normal muscle tone and reflexes. His pupillary responses were equal and reactive bilaterally. The patient weighed 14 kg, and all other aspects of his systemic examination, including vital signs, were unremarkable.

## 3 Diagnostic Investigations and Differential Diagnosis

Initial non‐contrast computed tomography (CT) imaging of the brain revealed a well‐demarcated, hypodense, rounded lesion within the posterior cranial fossa, raising suspicion for a posterior fossa tumor, such as a pilocytic astrocytoma. To clarify this finding and better characterize the lesion, a subsequent magnetic resonance imaging (MRI) scan was performed.

**FIGURE 1:**
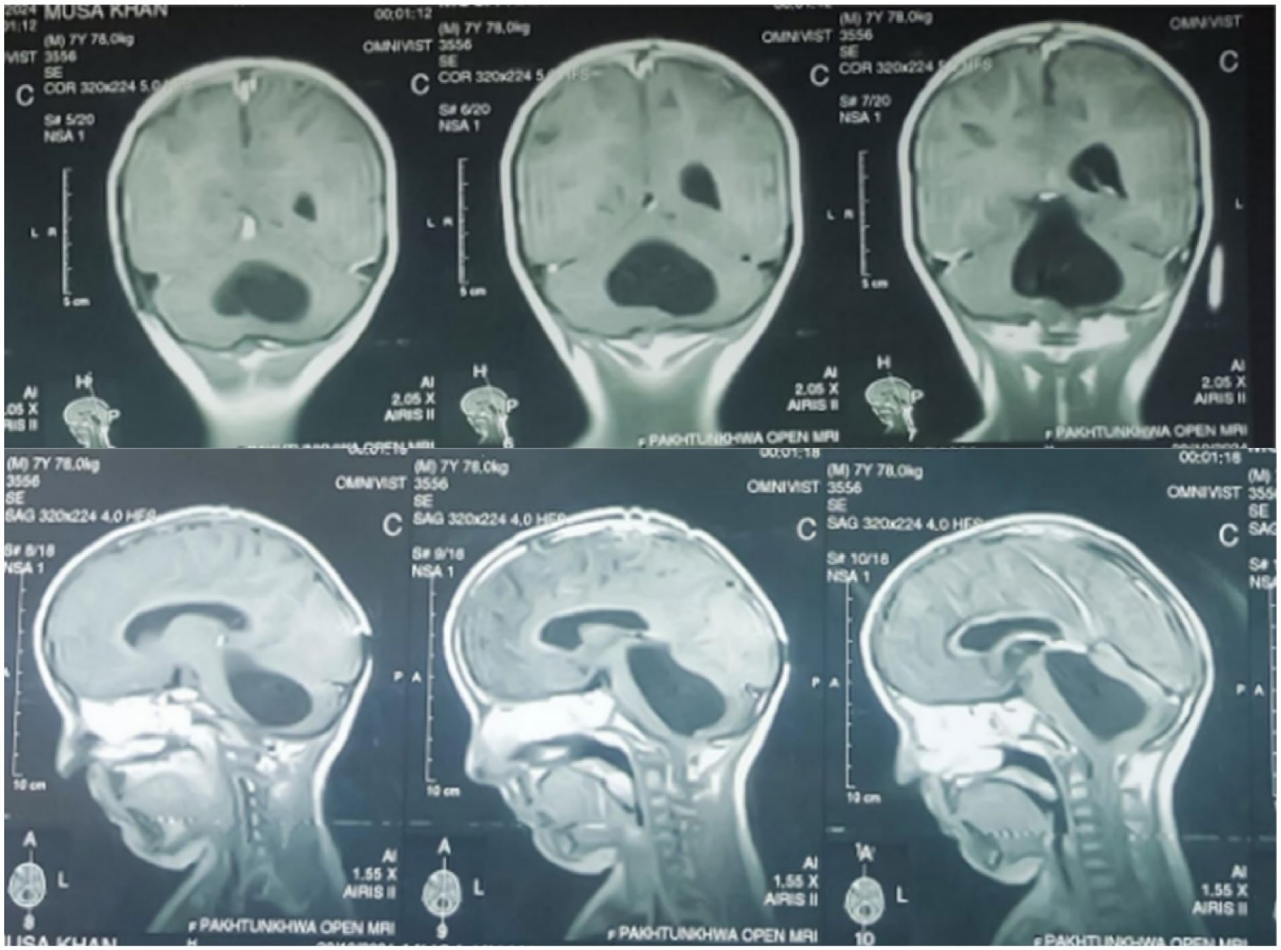
Pre‐operative MRI Brain showing hypointense lesion in the posterior cranial fossa on sagittal and coronal sections.

The MRI confirmed that the mass was not a cystic neoplasm but was, in fact, a markedly dilated fourth ventricle that appeared hypointense on imaging and occupied a significant volume in the posterior fossa, causing compression of the brainstem and cerebellum (Figure 1). Considering the patient's history of VP shunt placement for hydrocephalus, a definitive diagnosis of trapped fourth ventricle (TFV) was established.

## 4 Treatment, Outcomes, and Follow‐Up

For medical management, the patient was started on intravenous (IV) cefoperazone‐sulbactam (1 g twice daily) for infection prophylaxis, IV levetiracetam (250 mg twice daily, a dose selected based on institutional pediatric guidelines and the patient's clinical status) to prevent seizures, and IV dexamethasone (1 mL four times daily) to address periventricular edema.

After preoperative optimization and obtaining informed consent, the patient underwent surgical intervention. An open posterior fenestration was selected over an endoscopic approach to guarantee direct visualization and ensure complete lysis of any potential adhesions, as the patient's history of trauma and prior surgery presented a high risk of complex scarring that could compromise the safety and efficacy of an endoscopic‐only technique. The procedure involved a suboccipital craniotomy for open fenestration of the fourth ventricle with microsurgical arachnoid adhesiolysis aimed at restoring normal cerebrospinal fluid (CSF) outflow from the fourth ventricle to the cisterna magna. During the procedure, the pre‐existing supratentorial VP shunt was inspected, pumped, and confirmed to be patent and functional; it was therefore left in situ to continue managing supratentorial CSF drainage.

**FIGURE 2:**
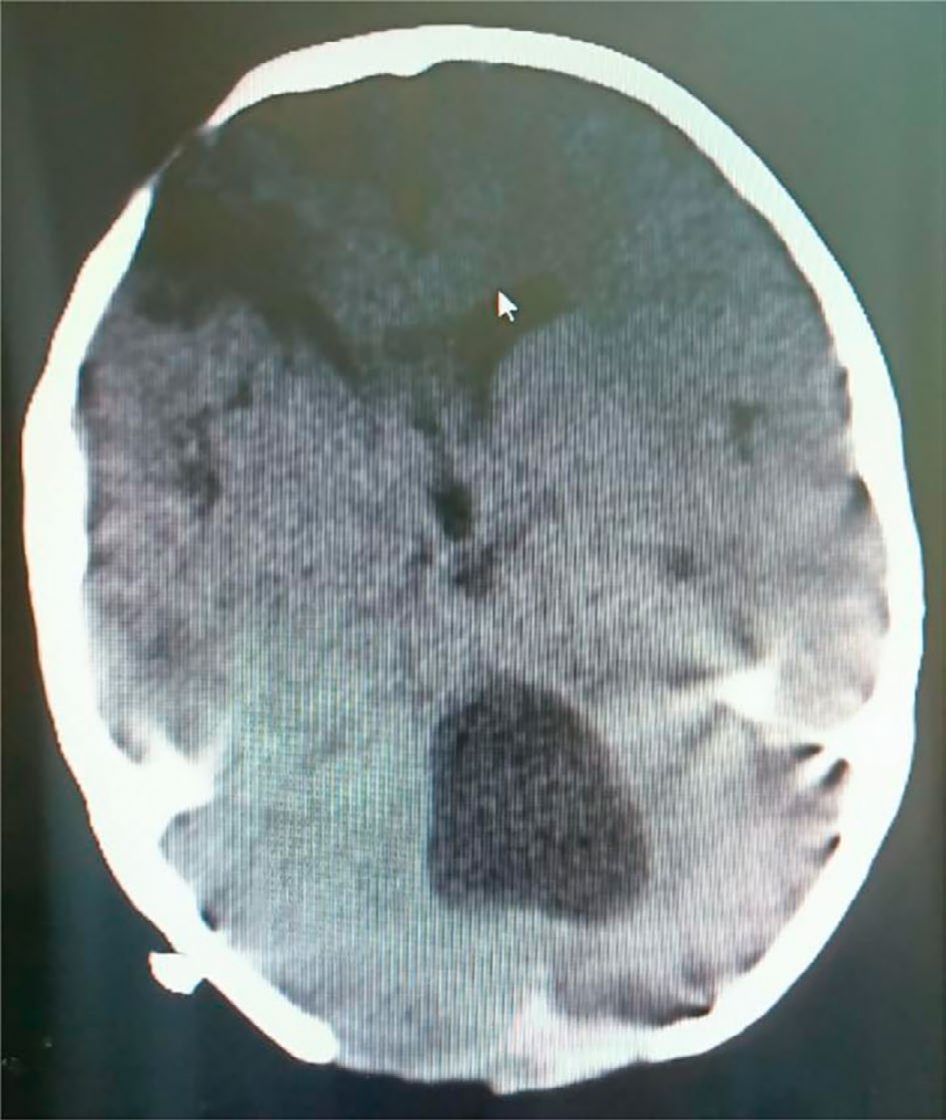
Postoperative CT scan showing craniotomy defects in the right occipital and right frontal bones, dilation of the ventricular chain with pneumocephalus, with VP shunt in place. The dilated fourth ventricle significantly reduced in size as the CSF flow became normal after fenestration.

Postoperatively, the patient had mild irritability and headache but experienced no seizures or other complications. Oral intake was successfully resumed 12 h after the procedure. A follow‐up CT scan performed on postoperative day 10 showed a significant reduction in the size of the fourth ventricle and confirmed that CSF dynamics had been restored (Figure 2). He continued to receive intravenous cefoperazone‐sulbactam and levetiracetam for seizure prophylaxis during his 10‐day hospitalization. At discharge, he was prescribed oral sodium valproate syrup (250 mg/5 mL twice daily) for seizure prophylaxis, with detailed instructions for administration and monitoring. At his one‐month follow‐up appointment, the patient was asymptomatic, showing a complete resolution of all his previous neurological deficits.

## 5 Discussion

This case highlights the diagnostic and therapeutic challenges of TFV, also known as an Isolated Fourth Ventricle (IFV), a rare but serious complication of shunted hydrocephalus. TFV develops when the aqueduct of Sylvius and the fourth ventricle's outflow pathways, the foramina of Magendie and Luschka, or the basal cisterns are blocked [12]. When pathology obstructs the CSF drainage pathway, CSF gradually accumulates, initially displacing the cerebellum before compressing the brainstem anteriorly [11]. While the exact mechanism leading to fourth ventricle entrapment remains incompletely understood, it is likely caused by the collapse of the lateral and third ventricles, leading to slit‐like ventricles [12, 13, 14]. Moreover, some studies propose that the combination of transtentorial herniation of a dilated posterior ventricle and upward aqueductal displacement from fourth ventricular enlargement can distort the aqueduct enough to isolate the fourth ventricle [15].

Implantation of a ventricular shunt is another potential cause [16]. However, it is most commonly seen in patients with a history of multiple shunt surgeries, post‐infectious hydrocephalus, or Youmans' isolated fourth ventricle (IFV) [11, 17]. The condition is particularly concerning in pediatric patients with a history of VP shunt placement, as altered CSF dynamics and post‐inflammatory adhesions can predispose to this complication [18].

Patients with an entrapped fourth ventricle exhibit a broad clinical spectrum, from asymptomatic cases detected incidentally on CT to fulminant posterior fossa syndrome manifesting as ataxia, altered consciousness, ocular motility disturbances such as diplopia or nystagmus, cognitive dysfunction, speech impairment, and cranial neuropathies [14]. Our patient's presentation with drowsiness, gait disturbances, and behavioral changes, along with imaging findings of an isolated, dilated fourth ventricle, underscores the importance of maintaining a high index of suspicion for TFV in shunted hydrocephalus patients who develop new neurological symptoms.

The pathophysiology of TFV involves a combination of aqueductal stenosis and compartmentalization of the ventricular system, often secondary to prior hemorrhage, infection, or trauma [19, 20]. In this case, the patient's history of a depressed skull fracture and subsequent VP shunt placement likely contributed to the development of arachnoid adhesions, disrupting normal CSF flow. The initial misdiagnosis of a posterior fossa tumor on CT imaging emphasizes the necessity of advanced neuroimaging, particularly MRI, in differentiating TFV from cystic lesions or neoplasms; however, CT‐based differentiation from cystic tumors is achievable through careful evaluation of density and imaging features [12]. MRI's superior soft‐tissue resolution allows for precise evaluation of ventricular anatomy and CSF flow dynamics, which is crucial for accurate diagnosis and surgical planning. Additionally, the combined metrizamide ventriculography and CT imaging also offers an effective diagnostic approach to distinguish between fourth ventricular entrapment and benign enlargement [14, 21]. Furthermore, advanced imaging modalities, including intraoperative MRI and neuronavigation systems, have markedly enhanced the precision of ventricular procedures, minimizing iatrogenic injury to adjacent neural tissues, leading to better surgical outcomes [22, 23].

Management of TFV remains controversial, with interventions including endoscopic third ventriculostomy (ETV), fourth ventricular shunting, open fenestration with adhesiolysis, or shunting with a Y connector [24, 25, 26, 27]. As illustrated in Table 1, prior studies reflect significant heterogeneity in etiology, clinical presentation, and therapeutic outcomes. In this case, we opted for an open posterior fenestration of the fourth ventricle. This decision was based on the high clinical suspicion of dense, inflammatory arachnoid adhesions resulting from the patient's history of trauma and prior surgery. Although not clearly visualized on imaging, such adhesions can compromise the success of an endoscopic approach. The open microsurgical technique was chosen to ensure direct visualization and complete lysis of any adhesions, thereby establishing a durable communication between the fourth ventricle and the cisterna magna. This approach also allowed for confirmation that the pre‐existing supratentorial VP shunt remained functional, which was left in situ to avoid the added complexity of a multi‐catheter system. The technique of open fenestration has been previously attempted in the literature, with reported success in managing fourth ventricular entrapment [19, 38, 41, 43].

**TABLE 1:** Summary of studies on trapped/isolated fourth ventricle: clinical presentation, management, and outcomes.

Study (Author, Year)	*N*	Predominant etiology	Intervention details	Key outcomes & complications
*Shunt‐based procedures*				
Dauda HA, et al., 2021 [28]	1	Post‐meningitic	Fourth Ventriculoperitoneal (FVP) shunt	Favorable clinical recovery
Hawkins Iii, et al., 1978 [6]	3	Shunt malfunction	Fourth ventricle shunt placement	Restoration of normal shunt function
James HE, et al., 1990 [13]	10	Post‐hemorrhagic	Fourth ventricle catheter connected to existing shunt	Symptom resolution noted
Lewis CS, et al., 2018 [29]	1	Post‐tumor resection	Fourth Ventriculopleural (VPL) shunt	Complete symptom resolution at 1‐year follow‐up
Colpan ME, et al., 2003 [30]	1	Post‐meningitic	Stereotactically‐guided FVP shunt	Gradual resolution of nystagmus and ataxia
Harrison HR, et al., 1982 [31]	1	Coccidioidal meningitis	FVP shunting	Favorable clinical recovery
Scotti G, et al., 1980 [32]	16	Various	FVP shunt	Clinical improvement reported in most patients
*Endoscopic procedures*				
Karachi C, et al., 2003 [33]	3	Idiopathic	Endoscopic Third Ventriculostomy (ETV)	All patients became symptom‐free and remained stable at 3‐year follow‐up
Raouf A, et al., 2013 [34]	13	Various	Endoscopic aqueductoplasty with stenting	High rate of stent migration (5/8) requiring reintervention
Ogiwara H, et al., 2013 [35]	9	Various	Endoscopic stent insertion	Multiple reinterventions were required due to catheter‐related issues
Fritsch MJ, et al., 2004 [36]	18	Various	Endoscopic aqueductoplasty ± stenting	7/18 patients (39%) required reoperation due to restenosis
Sansone JM, et al., 2005 [37]	9	Various	Endoscopic aqueductoplasty via foramen magnum	All patients improved; 1 required stenting for recurrent stenosis
Schulz M, et al., 2012 [38]	19	Various	Endoscopic aqueductoplasty or fenestration	27% full symptom resolution; 68% partial resolution. Complications in 5/19 patients (26%)
Gallo P, et al., 2012 [39]	18	Various	Endoscopic aqueductoplasty with stenting	All patients improved; 2 experienced transient cranial nerve (CN) deficits
Cinalli G, et al., 2006 [26]	7	Various	Endoscopic aqueductoplasty ± stenting	Restenosis occurred in 2 patients who did not receive a stent
Sagan LM, et al., 2006 [40]	5	Post‐hemorrhagic	Endoscopic aqueductal stent placement	All patients improved; 1 developed transient CN deficits
*Open microsurgical fenestration*				
Villavicencio AT, et al., 1998 [41]	6	Various	Suboccipital craniectomy + open fenestration	5/6 patients (83%) remained symptom‐free long‐term
Pang D, et al., 2005 [42]	1	Post‐shunt	Suboccipital craniectomy + C1 laminectomy	Early recovery of cranial nerve function
Udayakumaran S, et al., 2011 [43]	12	Various	Posterior fossa craniectomy + decompression	All patients showed clinical improvement
Tyagi G, et al., 2020 [44]	11	TB meningitis, trauma	Open posterior fenestration + arachnoid dissection	9/11 (82%) had symptomatic relief; 8/11 (73%) showed radiological improvement
*Comparative or mixed cohort studies*				
Teo C, et al., 1999 [45]	16	Various	Shunting (*n* = 8) vs. Endoscopy (*n* = 8)	Shunt group had a significantly higher revision rate (50%) compared to the endoscopy group
Salman R, et al., 2022 [46]	64	Post‐hemorrhagic	Conservative vs. Surgery (Shunt or Endoscopy)	No change with conservative therapy. Surgical cases showed improvement (25% normalized FV size)
El Damaty A, et al., 2020 [47]	21	Post‐hemorrhagic	FVP Catheters (*n* = 18) vs. Endoscopy (*n* = 2)	All treated patients improved; imaging confirmed FV decompression
Mohanty A, et al., 2018 [48]	25	Various	Endoscopic stents (*n* = 19) vs. FVP shunt (*n* = 6)	FVP shunt resulted in better reduction of FV size; stent migration was a common issue
Pomeraniec IJ, et al., 2016 [49]	8	Post‐hemorrhagic	Conservative (*n* = 5) vs. Surgery (*n* = 3)	Surgical group required repeated revisions; conservative group remained stable

This approach offers the advantage of direct visualization and lysis of adhesions, reducing the risk of re‐occlusion [44]. The patient's rapid postoperative recovery and resolution of symptoms further validate the efficacy of this technique. However, long‐term follow‐up is essential, as recurrent adhesions or shunt dysfunction may necessitate additional interventions. A limitation of this report is the relatively short follow‐up period of 1 month. While the patient's clinical and radiological outcomes were excellent at this time point, the potential for late recurrence due to the reformation of scar tissue necessitates continued long‐term surveillance.

Prophylactic measures, including antibiotics and antiepileptics, played a supportive role in minimizing perioperative risks. Additionally, the use of dexamethasone helped reduce periventricular edema, while levetiracetam provided seizure prophylaxis, particularly important given the patient's history of altered consciousness. The favorable outcome in this case underscores the importance of a multidisciplinary approach, which integrates neurosurgical expertise with meticulous postoperative care.

## 6 Conclusion

In conclusion, TFV is a rare but potentially life‐threatening complication of shunted hydrocephalus that requires prompt recognition and intervention. This case highlights the importance of recognizing clinical signs of TFV, underscores the value of MRI in making an accurate diagnosis, and demonstrates that open surgical fenestration remains an effective treatment strategy, particularly in cases where dense arachnoid adhesions are suspected and direct microsurgical management is preferred.

Prompt management of fourth ventricular entrapment is critical to alleviate symptoms of elevated intracranial pressure and life‐threatening brainstem compression, including cranial neuropathies, dysphagia, and dysarthria. Early intervention not only prevents permanent neurological deficits but also preserves developmental progress in pediatric patients while optimizing surgical prognosis [11, 47]. While this case demonstrates an excellent short‐term result, future studies should focus on optimizing surgical techniques and evaluating long‐term outcomes to refine management protocols for this challenging condition.

## Author Contributions

**Muhammad Hamza:** conceptualization, data curation, formal analysis, methodology, project administration. **Muhammad Ibrahim:** conceptualization, data curation, visualization, writing – original draft, writing – review and editing. **Ihsan Ullah:** conceptualization, data curation, formal analysis, investigation. **Rehmat Farhaj:** methodology, project administration, writing – original draft, writing – review and editing. **Fazeela Bibi:** writing – original draft, writing – review and editing. **Khalil El Abdi:** conceptualization, project administration, writing – original draft, writing – review and editing. **Bilal Aslam:** resources, software, writing – original draft, writing – review and editing. **Vohra Maham Hassan:** conceptualization, data curation, writing – original draft, writing – review and editing. **Said Hamid Sadat:** resources, software, visualization, writing – original draft.

## Funding

The authors have nothing to report.

## Ethics Statement

This case report was conducted in accordance with the ethical standards of our institution and the Helsinki Declaration. Per institutional policy, formal ethics committee approval is not required for a retrospective case report involving a single patient, provided patient consent for publication is obtained and all identifying information is protected.

## Consent

Written informed consent was taken from the patient to publish this Manuscript.

## Conflicts of Interest

The authors declare no conflicts of interest.

## Data Availability

The data was taken from a patient who presented to our hospital; all data and references are publicly available on databases such as Pub‐med and Google Scholar.
